# Two Cases of Right Atrial Thrombus in Transit: Evaluation, Echocardiography, and Management

**DOI:** 10.7759/cureus.93496

**Published:** 2025-09-29

**Authors:** Kayla M Knuf, Joseph Mellen, Theodor Danciu, Matthew Smith

**Affiliations:** 1 Anesthesiology, Brooke Army Medical Center, San Antonio, USA; 2 Anesthesiology, Uniformed Services University of the Health Sciences, Bethesda, USA

**Keywords:** acute pulmonary embolism, acute right ventricular failure, catheter-directed thrombectomy, echocardiography in icu, intracardiac thrombus

## Abstract

Intracardiac thrombus can present across a broad clinical spectrum, from incidental findings to life-threatening cardiogenic shock. Management is determined by both the patient’s condition and the thrombus characteristics. Catheter-directed thrombectomy has emerged as a viable treatment option for selected patients. We present two cases of right atrial thrombus managed with catheter-based intervention, accompanied by a detailed discussion of the clinical evaluation, echocardiographic imaging, and management strategies in critically ill patients.

## Introduction

Intracardiac thrombi represent a critical cardiovascular emergency, particularly when identified in association with acute pulmonary embolism or cardiogenic shock. These “clots in transit” are highly mobile and carry a significant risk of embolization, acute right ventricular (RV) failure, and sudden hemodynamic collapse if not promptly treated [1]. In the general population, autopsy data indicate a prevalence of intracardiac thrombus of 7.2% among in-hospital deaths, with right and left atrial thrombi occurring at similar rates [2]. 

In recent years, catheter-directed thrombectomy has become a minimally invasive and potentially life-saving intervention for appropriately selected patients [3]. Here, we describe two cases of right atrial thrombus managed with catheter-based techniques. Through these cases, we highlight the diagnostic value of echocardiography, the principles guiding intervention, and the procedural considerations, including imaging selection, sedation strategy, and RV risk assessment, which shape the management of this high-risk condition.

## Case presentation

Case 1

**Figure 1:**
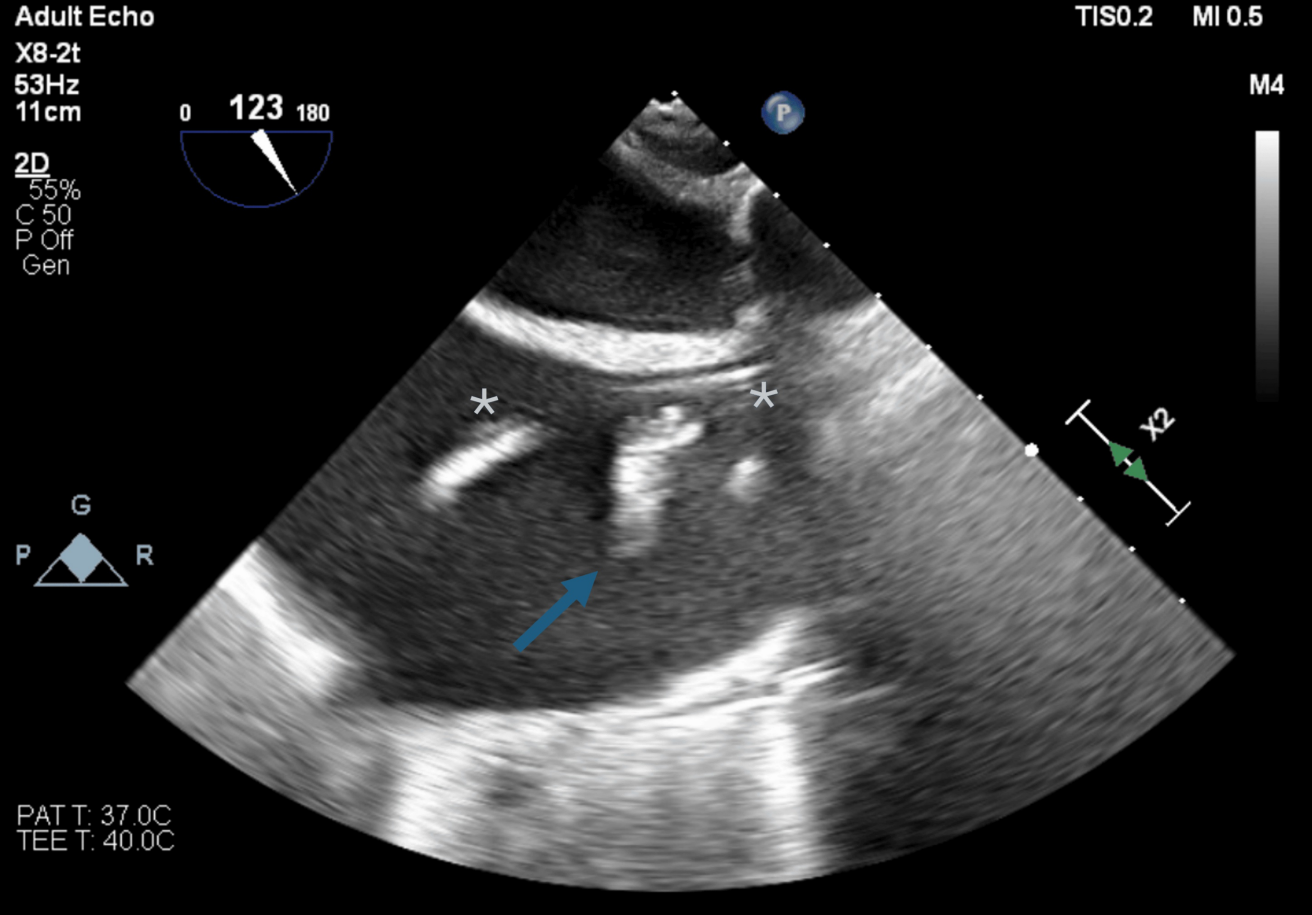
Case 1: Midesophageal bicaval view, focused on the thrombus (arrow) adhered to the pulmonary artery catheter (asterisks).

A 56-year-old man presented with acute decompensated heart failure secondary to porcine valve dysfunction. The patient underwent aortic valve replacement and ascending aortic graft replacement. The case was complicated by extensive scar tissue, resulting in prolonged time on cardiopulmonary bypass and difficulty weaning. The patient’s postoperative course was complicated by acute renal failure requiring continuous renal replacement therapy (CRRT), pneumonia, and severe coagulopathy. On postoperative day three, the patient went into atrial fibrillation with a rapid ventricular rate. He could not be mechanically or chemically cardioverted despite several attempts. Due to worsening hemodynamics, he was placed on venovenous extracorporeal membrane oxygenation (V-A ECMO). The patient improved and was extubated on postoperative day five, and CRRT was discontinued on postoperative day nine. On postoperative day 14, he was decannulated from V-A ECMO, assisted by real-time transesophageal echocardiography (TEE). During echocardiographic evaluation, a right atrial thrombus was identified adhering to the pulmonary artery catheter and superior atriocaval junction (Figure 1). Due to concern for future obstruction, the patient underwent a right atrial thrombectomy with interventional radiology on postoperative day 15. He arrived with an endotracheal tube in place, avoiding the need for induction of anesthesia and allowing real-time TEE guidance during the procedure. He arrived with an endotracheal tube in situ, avoiding the need for induction of anesthesia and facilitating real-time TEE guidance during the procedure. Ultimately, the thrombus was removed via suction thrombectomy, and the patient was extubated the following day. Biventricular function continued to improve, but septic shock and vasoplegia complicated the remainder of his hospital course. He eventually stabilized, began his recovery phase, and was discharged on hospital day 57.

Case 2

**Figure 2:**
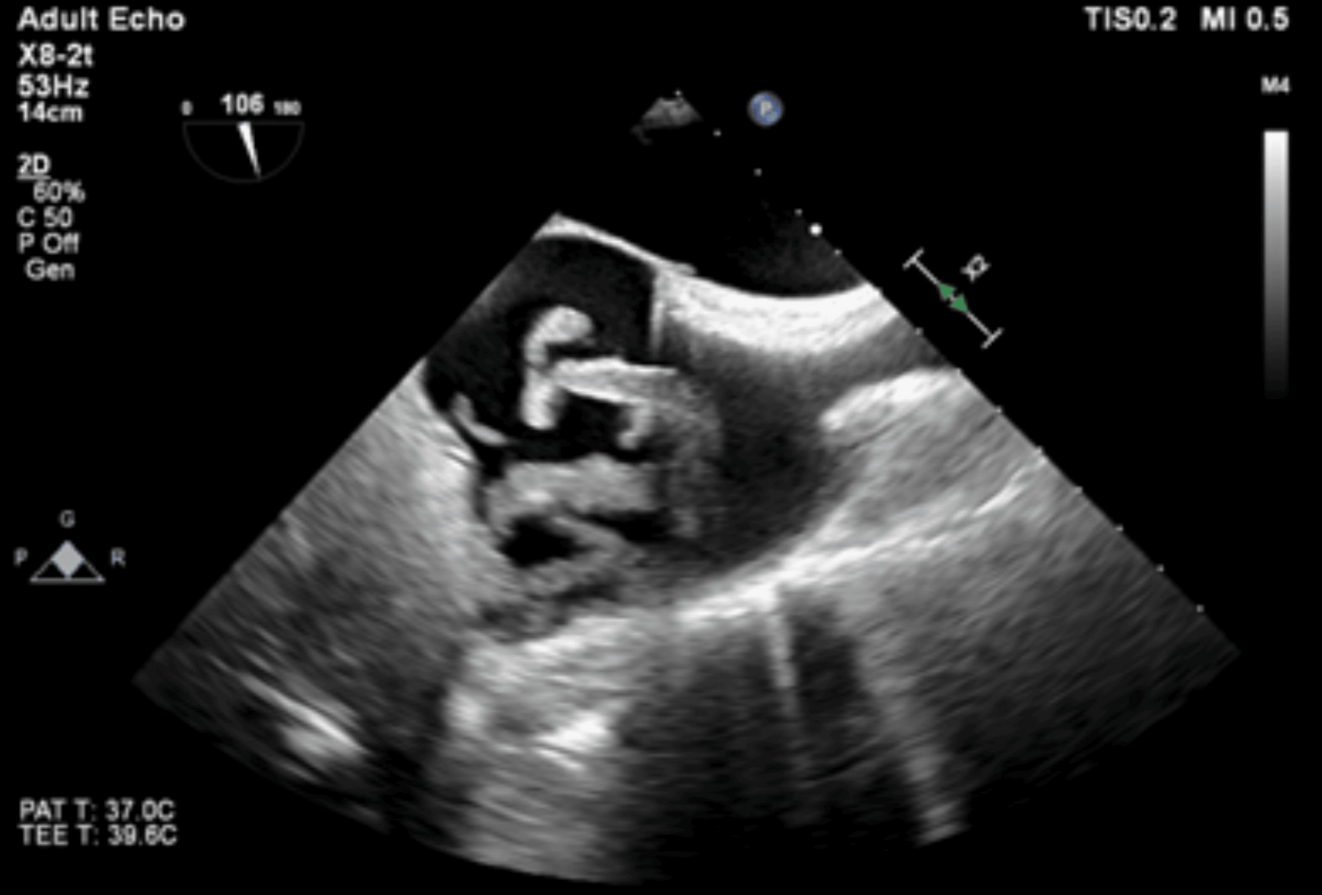
Case 2: A midesophageal bicaval view of the clot removed demonstrating a multi-lobular unattached thrombus in the right atrium.

**Figure 3:**
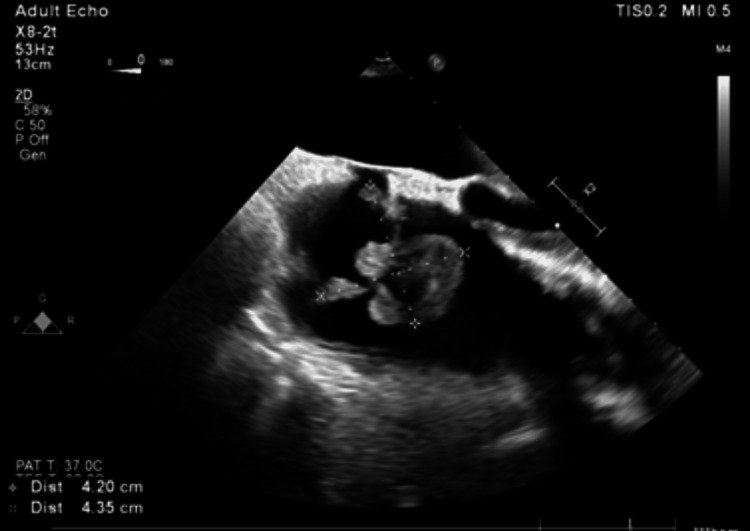
Case 2: A midesophageal four chamber view of a 4.2 cm by 4.3 cm clot removed

A 67-year-old man with a history of daily ethanol use was found unconscious at home during a wellness check. He was intubated at the scene and brought to the hospital for further evaluation. Initial workup demonstrated a subarachnoid hemorrhage and multiple cerebral infarcts with midline shift, intrapulmonary fluid consistent with aspiration, and left-sided rib fractures with associated hemopneumothorax. Bedside echocardiographic examination demonstrated a mobile right atrial thrombus and marked RV strain with tricuspid annular plane systolic excursion (TAPSE) less than 1.7 cm and an RV S Prime (S’) via tissue Doppler interrogation of less than 10 cm/s. The patient’s family requested that all life-saving efforts continue, so the decision was made to move emergently to the interventional radiology suite for catheter thrombectomy. Anesthesia support was requested, and the case was performed with intraoperative TEE. See Figures 2, 3 for TEE imaging during this case. The patient had near-full RV recovery after the procedure, but continued to have marked vasoplegia from multisystem organ failure. Unfortunately, his neurological prognosis remained poor, and he was ultimately placed on comfort care measures at the request of his family. 

## Discussion

Intracardiac thrombus is a potentially fatal emergency that can present variably, either forming in situ or appearing as clots in transit from the venous system. Right heart involvement can cause profound hemodynamic compromise. In the intensive care unit (ICU), pulmonary embolism (PE) prevalence varies, averaging 2% to 5% [4, 5]. While smaller, chronic emboli may be tolerated, acute, large PEs cause abrupt increases in RV afterload, precipitating hypoxemia, decreased cardiac output, and acute RV collapse.

Identifying risk factors for RV collapse can guide the next clinical steps. Among these, echocardiography is widely adopted because of its increasing versatility and ubiquity in the ICU. Specifically, features such as RV S’, TAPSE, and TAPSE/RV systolic pressure (RVSP) can help triage patients who may benefit from more invasive therapies [6]. Transthoracic echocardiography (TTE) is often preferred for quantifying RV systolic function because the angle of interrogation is preferable to TEE images. In addition, TTE is relatively low-risk and non-invasive and can be performed rapidly at the bedside. Both TAPSE and RV S’ are measures of global RV systolic function, with lower limits of normal typically noted as 1.7 cm and 10 cm/s, respectively. In Case 2, the patient had an obvious thrombus in transit necessitating intervention, but the reduced TAPSE and RV S’ suggest impending RV failure without resolution of the clot burden. 

In addition to assisting with diagnosis and classification, TTE can also allow for procedural guidance during catheter-directed thrombolysis and evaluate the resolution of the clot following the procedure. Additional invasive measurements can be useful as well. These include pulse pressure contour analysis from arterial pressure waveforms and central venous and pulmonary artery pressure analysis (e.g., pulmonary artery pulsatility index). Finally, the use of agitated saline or other contrast agents can help determine the extent of the thrombus, look for septal defects, and evaluate RV function. No measurements of RV function have demonstrated superiority. 

Right atrial thrombi carry a high mortality risk if untreated. Historically, treatment options have included systemic thrombolysis and surgical removal. However, in hemodynamically unstable patients, surgical treatment may have a worse mortality [7]. With this in mind, catheter-directed thrombectomy has recently been increasingly utilized in high-risk patients and/or patients who are not surgical candidates [8, 9]. There have been no randomized controlled trials to assist in treatment selection between surgical, systemic, and catheter-directed options. Although this strategy has been described only in select cases, existing case reports show success in patients of similar hemodynamic instability as the two reported here. 

The approach to sedation management is determined by patient stability. When feasible, spontaneous, negative-pressure ventilation with minimal sedation is preferred over general anesthesia, which can precipitate RV failure. Induction of anesthesia, which can cause periods of hypoxemia, hypercarbia, decreased coronary perfusion pressure, and increased pulmonary pressures, can lead to acute RV failure, which can be fatal. Induction of general anesthesia and intubation risks a dose-dependent reduction in preload and RV inotropy from sedative-hypnotic agents, as well as the potential for tachycardia. If a general anesthetic is necessary for management, preparation is key to a safe induction in this patient population, including invasive blood pressure monitoring, adequate vascular access, and the immediate availability of vasopressors and inotropic medications. Cannulation for ECMO should be implemented for patients with a clinical picture concerning impending cardiovascular collapse. The safest induction strategy involves slow titration of agents, selecting sedative-hypnotics with minimal cardiovascular depression (such as etomidate), maintaining vigilant hemodynamic monitoring, and carefully avoiding coughing or straining that could precipitate thrombus embolization. 

Intraoperative imaging guidance during clot retrieval requires a combination of modalities. Fluoroscopy is used to obtain access and evaluate the filling of the right atrium and ventricle, but cannot reliably guide the retrieval device to the clot, assess the clot burden, or determine the degree of improvement after retrieval. The aspiration devices are readily guided via contrast fluoroscopy to occluded vessels where the relatively fixed PEs are easily targeted. In contrast, mobile clots in the right-sided cardiac chambers will oscillate with cardiac motion, are prone to tangling in cardiac structures, and can migrate into the pulmonary vasculature; therefore, TEE is often required. TEE provides live assessment of worsening clot burden, early warning of forward migration, and constant monitoring of RV function. Retrieval devices are directed to the clot surface via TEE. Evaluation of clot engagement and removal can be viewed in real time while simultaneously assessing cardiac and valvular function. The devices can impair filling, mechanically tangle within the tricuspid apparatus, or interact with other devices already present (e.g., pacing wires). In the case of aspiration pumps, the onset of high rotational speeds can suck down on adjacent tissues or reduce RV stroke volume. Post-procedure TEE should evaluate for success via examination of the cardiac chambers for residual clot, along with any cardiac or valvular dysfunction. While TEE is superior to TTE when evaluating right atrial thrombi, there is an important place for surface echocardiography in these patients. Due to the nature of the disease process and risks with sedation and positive pressure ventilation, there are clinical situations where TEE may be unsafe. Becoming facile with TTE can aid in the diagnosis and treatment of these patients. TTE views for right atrial thrombus identification and characterization include parasternal short-axis, RV inflow, apical four-chamber, and subxiphoid views [10]. Surface echo can also allow for procedural guidance during catheter-directed thrombolysis, as well as evaluation for resolution of the clot following the procedure.

Both presented patients had mobile right heart thrombi, a finding almost exclusively associated with suspected or confirmed PE [11]. Identifying anatomical relationships with pacemaker wires, valves, or the interatrial septum can help confirm the diagnosis. Thrombi typically appear as well-defined, echo-dense masses distinct from the endocardium, though differentiation based on imaging alone can remain challenging [12]. Clinical context is therefore paramount.

## Conclusions

Right atrial thrombi in critically ill patients require urgent diagnosis and coordinated multidisciplinary intervention due to the substantial risk of acute RV failure and fatal PE. The novelty of these cases lies in the coordinated use of TEE and catheter-directed thrombectomy as a valuable therapeutic option in two patients where surgical removal was not feasible or the patient was not a candidate. These cases add to the currently small pool of data in which catheter systems are increasingly utilized; more importantly, within this pool, we demonstrate the value of echocardiography at all stages of non-surgical treatment. As optimal outcomes hinge on early echocardiographic recognition, deliberate procedural planning, and real-time imaging, ongoing advancements in these areas will continue to shape treatment strategies and enhance survival for this high-risk population. 
